# Prenatal Diagnosis of WAGR Syndrome

**DOI:** 10.1155/2015/928585

**Published:** 2015-10-28

**Authors:** Berrin Tezcan, Philip Rich, Amarnath Bhide

**Affiliations:** ^1^Department of Women's Health, St George's University Hospital NHS Foundation Trust, Blackshaw Road, Tooting, London SW17 0QT, UK; ^2^Department of Neuroradiology, St George's University Hospital NHS Foundation Trust, Blackshaw Road, Tooting, London SW17 0QT, UK

## Abstract

Wilm's tumour, aniridia, genitourinary abnormalities, and mental retardation (WAGR) syndrome is a rare genetic disorder with an estimated prevalence of 1 in 500,000 to 1 million. It is a contiguous gene syndrome due to deletion at chromosome 11p13 in a region containing WT1 and PAX6 genes. Children with WAGR syndrome mostly present in the newborn/infancy period with sporadic aniridia. The genotypic defects in WAGR syndrome have been well established. However, antenatal ultrasonographic presentation of this syndrome has never been reported. Prenatal diagnosis of this condition is possible in some cases with careful ultrasound examination of classical and nonclassical manifestations of this syndrome. The key point for this rare diagnosis was the decision to perform chromosomal microarray analysis after antenatal diagnosis of absent corpus callosum and absent cavum septum pellucidum, as this finding mandates search for potentially associated genetic disorders. We report a case of WAGR syndrome diagnosed prenatally at 29-week gestation. The diagnosis of the anomaly was based on two- and three-dimensional ultrasound as well as fetal MRI scan and microarray analysis. The ultrasonographic findings included borderline ventriculomegaly, absent corpus callosum, and absent cavum septum pellucidum. Cytogenetic results from the amniotic fluid confirmed WAGR syndrome. Parental karyotype was normal, with no evidence of copy number change, deletion, or rearrangement of this region of chromosome 11.

## 1. Case Presentation

We report a case of 30-year-old fit and well Asian (Indian ethnicity) woman in her second pregnancy, with a BMI of 26. The couple were nonconsanguineous.

**Figure 1:**
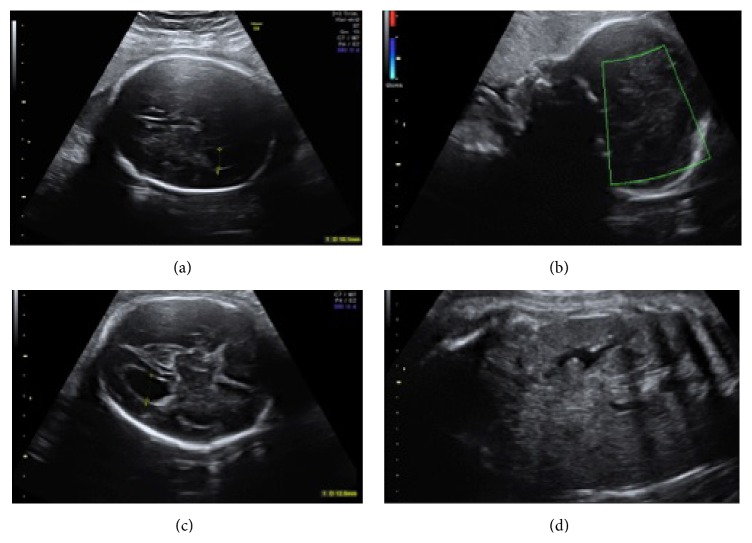
Ultrasound images of absent corpus callosum, mild ventriculomegaly, and enlarged kidney. (a) US image of absent cavum septum pellucidum. (b) US image of absent corpus callosum. (c) US image of ventriculomegaly. (d) US image of enlarged kidney.

In the current pregnancy, her 11–14 weeks' scan revealed no major structural abnormalities in the fetus and the risk for Down syndrome was low (1 : 50000) on combined screening. A routine anomaly scan at 20 weeks was also reported as normal. She was diagnosed with gestational diabetes at 16 weeks that was diet controlled and was offered serial growth scans from 28 weeks. A growth scan at 29 weeks showed reduced growth, and in view of this the woman was referred to the Fetal Medicine Unit. Scan performed by the fetal medicine specialist showed borderline bilateral ventriculomegaly, absent corpus callosum, and absent cavum septum pellucidum. The amniotic fluid volume was mildly reduced. All the other systems appeared normal (Figure 1). The couple were counselled regarding the possibility of genetic or chromosomal causes, and they opted for an amniocentesis. A fetal MRI was also organised for further assessment of the brain. Microarray result indicated a copy number loss for the short arm of chromosome 11 with break points between 11p12 and 11p14.1. Deletion of this region indicates WAGR 11p13 deletion syndrome.

Another detailed ultrasound scan was performed before the late termination. In addition to the above findings, large kidneys visualised. Measurements of both kidneys' volumes were above the 97th centile for gestational age. There was no evidence of hydronephrosis (Figure 1).

## 2. Results

### 2.1. Fetal Brain MRI

**Figure 2:**
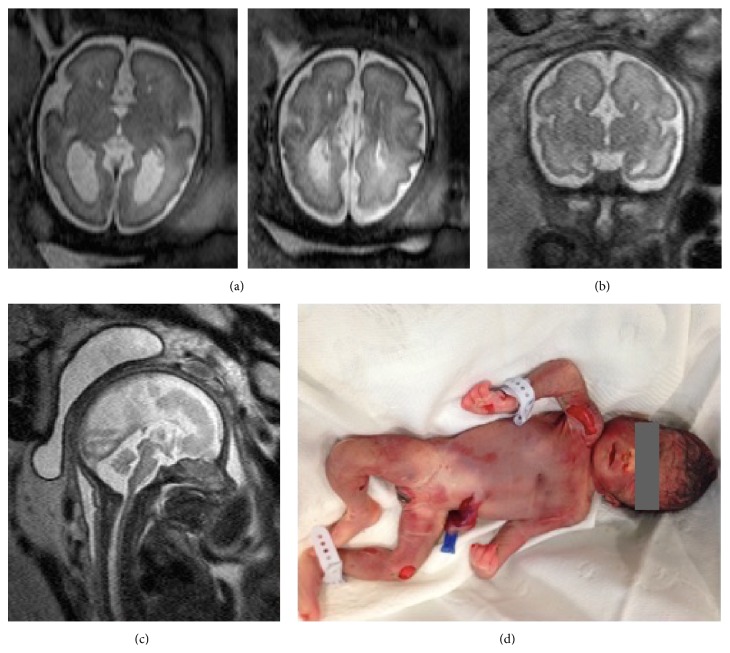
Fetal MRI images of absent corpus callosum and the picture of the newborn. (a) Axial scans at the level of the atria and bodies of the lateral ventricles show bilateral posterior ventriculomegaly. Anteriorly the lateral ventricles are narrow and parallel. This is the appearance of colpocephaly which is characteristically associated with ACC. (b) Coronal MRI scan at the level of third ventricle shows agenesis of the corpus callosum. The bodies of the lateral ventricles have a lateral convexity in the coronal plane and a medial concavity due to the presence of Probst's white matter bundles. This is the typical appearance in callosal agenesis. (c) Midsagittal MRI scan confirms absence of the corpus callosum. (d) Photograph taken in the postnatal period.

Agenesis of corpus callosum was confirmed. There was associated colpocephaly causing widening of the lateral ventricles posteriorly. Elsewhere the brain was normal in appearance. Sulcation and linear measurements were within expected limits for gestational age (Figure 2). Midsagittal MRI scan confirmed absence of the corpus callosum. Coronal MRI scan at the level of the third ventricle showed agenesis of the corpus callosum. The bodies of the lateral ventricles had a lateral convexity in the coronal plane and a medial concavity due to the presence of Probst's white matter bundles. This was in keeping with the typical appearance in callosal agenesis. Axial scans at the level of the atria and bodies of the lateral ventricles showed bilateral posterior ventriculomegaly. Anteriorly the lateral ventricles were narrow and parallel. This was the appearance of colpocephaly which is characteristically associated with ACC (Figure 2).

### 2.2. Fetal Echocardiogram

Mild to moderate biventricular hypertrophy with a small rim of pericardial fluid by right ventricle wall was seen, but the heart was structurally normal.

### 2.3. Cytogenetic Investigations

#### 2.3.1. Fetal Amniotic Fluid

 Karyotyping: QF-PCR analysis showed a normal compliment of chromosomes 13, 18, and 21 and an XX (female) chromosome complement.
 Microarray: it was as follows: Arr 11p14.1p12(30,863,700-38,018,632) x1

Genome-wide array analysis indicated a copy number loss for the short arm of chromosome 11 with breakpoints between 11p12 and 11p14.1. This result is consistent with a deletion of approximately 7.2 Mb. Deletion of this region is associated with WAGR 11p13 deletion syndrome (Wilms' tumor, aniridia, genitourinary malformation, and mental retardation syndrome). This is a contiguous gene deletion syndrome due to haploinsufficiencies of the gene in this region, including WT1 and PAX6, both of which were deleted in this patient [1–3].

## 3. Outcome

In view of the microarray result showing a deletion in the short arm of chromosome 11 associated with WAGR syndrome and the MRI brain confirming ultrasound findings, the parents were counselled regarding the outcome and parents decided to terminate the pregnancy.

A feticide procedure was performed at 31 weeks and this was followed by medical termination of pregnancy. The parents were offered a postmortem examination but it was declined. There were no obvious external structural anomalies on postnatal examination (Figure 2). An appointment with the clinical geneticist was organised.

Both parents agreed to cytogenetic testing and qPCR and FISH analysis showed normal results indicating that there was no evidence of copy number change, deletion, or rearrangement of this region of chromosome 11 suggesting that this genetic change was de novo in origin. Hence, the risk of recurrence of deletion seen in this fetus in a subsequent pregnancy was deemed to be low.

## 4. Discussion

WAGR syndrome is rare with an estimated prevalence of 1 in 500,00 to 1 per million. Most cases are identified in infancy due to sporadic aniridia, 30% of whom test positive for the WAGR deletion. It is characterised by Wilms' tumour, and children with the condition should receive regular renal surveillance until the age of 6–8 years and thereafter remain under renal surveillance because of the 40% risk of late onset nephropathy over the age 12. Vaginal and uterine malformation may also be present in affected girls, and streak ovaries might increase their risk for gonadoblastoma [1, 2].

Genomic copy number imbalances are being identified as one of the important causes of behavioral abnormalities and intellectual disability. The typical deletion in WAGR syndrome includes the WT1 and PAX6 genes, but larger deletions can be associated with obesity and neurobehavioral abnormalities [3].

One recent study investigated two patients, who showed the chromosomal deletions involving 11p13. First patient, diagnosed with a 8.6 Mb deletion of chr11p14.1p12: 29,676,434–38,237,948, showed a phenotype typical of WAGR syndrome and had significant learning disabilities and autistic behaviors. Second patient had a larger deletion in 11p14.1-p12 which was separated into two regions, that is, a 10.5 Mb region of chr11p13p12: 32,990,627–43,492,580 and a 2.2 Mb region of chr11p14.1: 29,195,161–31,349,732. As a consequence, 1.6 Mb region of the WAGR syndrome region which was critical was intact between the two deletions. The second patient has showed no symptom of WAGR syndrome and no autistic behaviors. Therefore, the region responsible for severe developmental delay and autistic features on WAGR syndrome narrowed down to the region remaining intact in the second patient. Therefore, this study suggested that haploinsufficiencies of PAX6 or PRRG4 included in this region were candidate genes for severe developmental delay and autistic features characteristic of WAGR syndrome [4].

In a published case review of 54 cases, Fischbach et al. [5] studied patients with WAGR syndrome aged from 7 months to 42 years and demonstrated a wide range of clinical signs of this syndrome. They reported enlarged ventricles in two cases and agenesis of the corpus callosum in two further cases. In this study, 17 out of 54 cases showed associated structural abnormalities and diagnosis of these structural abnormalities antenatally should alert the clinician. These structural abnormalities were enlarged ventricles (2 cases), microcephaly (1 case), agenesis of corpus callosum (2 cases), periventricular heterotopia (1 case), microcephaly (1 case), cerebellar hypoplasia (1 case), renal cysts (1 case), unilateral renal agenesis (1 case), hypoplastic kidney (1 case), tetralogy of Fallot (1 case), VSD (2 cases), ASD (1 case), micrognathia (1 case), and cleft palate (1 case).

WAGR syndrome is a rare genetic disorder and prenatal diagnosis of this syndrome has never been reported. It is possible to diagnose some cases with careful ultrasound examination of classical and nonclassical manifestations of this syndrome. The key point for this rare diagnosis was the decision to perform chromosomal microarray analysis after antenatal diagnosis of absent corpus callosum and absent cavum septum pellucidum, as this finding mandates search for potentially associated genetic disorders. Through this case report we are able to diagnose this rare genetic syndrome from the antenatal period and offer parents information regarding the syndrome and help them to make their informed decision and also to clarify the risk of recurrence in future pregnancies.
